# Turgor Pressure and Possible Constriction Mechanisms in Bacterial Division

**DOI:** 10.3389/fmicb.2018.00111

**Published:** 2018-01-31

**Authors:** Masaki Osawa, Harold P. Erickson

**Affiliations:** Department of Cell Biology, Duke University Medical Center, Durham, NC, United States

**Keywords:** ftsZ, bacterial division, tubulin, cytokinesis, turgor pressure

## Abstract

Bacterial cytokinesis begins with the assembly of FtsZ into a Z ring at the center of the cell. The Z-ring constriction in Gram-negative bacteria may occur in an environment where the periplasm and the cytoplasm are isoosmotic, but in Gram-positive bacteria the constriction may have to overcome a substantial turgor pressure. We address three potential sources of invagination force. (1) FtsZ itself may generate force by curved protofilaments bending the attached membrane. This is sufficient to constrict liposomes *in vitro*. However, this force is on the order of a few pN, and would not be enough to overcome turgor. (2) Cell wall (CW) synthesis may generate force by pushing the plasma membrane from the outside. However, this would probably require some kind of Brownian ratchet to separate the CW and membrane sufficiently to allow a glycan strand to slip in. The elastic element is not obvious. (3) Excess membrane production has the potential to contribute significantly to the invagination force. If the excess membrane is produced under the CW, it would force the membrane to bleb inward. We propose here that a combination of FtsZ pulling from the inside, and excess membrane pushing membrane inward may generate a substantial constriction force at the division site. This combined force generation mechanism may be sufficient to overcome turgor pressure. This would abolish the need for a Brownian ratchet for CW growth, and would permit CW to operate by reinforcing the constrictions generated by FtsZ and excess membrane.

## Introduction

The cytokinesis system of almost all bacteria and many archaea is based on a ring of the tubulin homolog FtsZ (Bi and Lutkenhaus, 1991), termed the Z ring, which recruits up to a dozen downstream proteins to achieve constriction of the membranes and cell wall (CW). FtsZ polymerizes into short protofilaments (∼150–300 nm) which further associate into the Z ring. In many bacteria the protofilaments are tethered to the plasma membrane (PM) by FtsA, which binds the C-terminal peptide of FtsZ and inserts its own C-terminal amphipathic helix into the PM (Pichoff and Lutkenhaus, 2005). FtsA also plays a role in recruitment of downstream molecules that remodel the peptidoglycan CW (Weiss et al., 1999).

An important question for cytokinesis is whether the invagination of the PM needs to overcome the turgor force. The turgor force is considerable, with estimates ranging from 0.3 atm (Deng et al., 2011) to 3 atm (Cayley et al., 2000) for *Escherichia coli*, and up to 20 atm for *Bacillus subtilis* (Whatmore and Reed, 1990). For comparison a racing bicycle tire has a pressure of about 10 atm. If cytokinesis has to overcome this turgor force it will need very high forces. Whether it does has been a question of controversy in the field, and even between the two present authors.

Erickson has suggested that bacterial cytokinesis might “cheat turgor pressure” by taking place completely within the high pressure environment (Erickson, 2017). This seems well-established for Gram-negative bacteria, where the turgor pressure is generated and sustained by the outer membrane plus the CW, and the periplasm is isoosmotic with the cytoplasm. Then both PM ingression and adding peptidoglycan to the inner surface of the CW to grow the septum take place without fighting turgor. Erickson argued that this is likely also true for Gram-positive bacteria. The strongest argument for this is that cryo EM shows a periplasmic space of ∼20 nm between the PM and CW for Gram-positive bacteria. If the periplasm were not isoosmotic, the PM should be pressed against the CW. Isoosmolarity would be achieved if the thicker CW itself functions as a semipermeable membrane, with a porosity excluding proteins and molecules larger than ∼1,000 Da.

On the contrary, Osawa notes several lines of evidence that the CW of Gram-positive bacteria is leakier than the CW plus outer membrane of Gram-negative bacteria. First, at least one study of the CW of *B. subtilis* proposed a sieving size of 2 nm, which corresponds to the diameter of a 20 kDa globular protein (Demchick and Koch, 1996).

Second, the Sec and Tat secretion systems of *B. subtilis* have been well-delineated for transport across the PM, but no dedicated system has been described for transport across the CW. The simplest mechanism would be that once transported across the PM, small proteins such as beta-lactamase (29 kDa), cutinase (24 kDa), and GFP (28 kDa) diffusely pass through the CW into the extracellular medium (Palva et al., 1982; Tjalsma et al., 2004; Brockmeier et al., 2006; Vrancken et al., 2007). In contrast Gram-negative bacteria have a “main terminal branch” for transport of secretory proteins across the CW and outer membrane. In fact, an *E. coli* strain K 12 that does not express this transporter virtually does not secrete the periplasmic proteins to the media (Pugsley et al., 1997).

Third, many bacteriocins, which are peptides and proteins up to ∼20 kDa, are known to attack the PM of various Gram-positive bacteria, which means they are able to cross the CW. For example cyclic antibacterial peptide Enterocin AS-48 (7 kDa), which has a compact globular structure, kills a wide variety of Gram-positive bacteria by damaging the PM (Grande Burgos et al., 2014). Phospholipase A2 (14 kDa) also kills actively growing Gram-positive bacteria by extensive degradation of phospholipid (Foreman-Wykert et al., 1999). Dysgalacticin (21.5 kDa), a heat-labile bacteriocin, can kill both growing and non-growing *Streptococcus pyogenes* (Gram-positive bacteria) (Swe et al., 2009).

These results suggest the possibility that ∼20 kDa molecules can diffusively pass through the CW of Gram-positive bacteria. If this is the case, how could Gram-positive bacteria maintain a periplasm isoosmolar with cytoplasm? One possibility is that negatively charged lipoteichoic acids tethered to the PM create a Donnan-like equilibrium that mitigates the difference of osmotic pressure between cytoplasm and periplasm. This has been suggested by two studies (Oku et al., 2009; Percy and Grundling, 2014). Quantitative analysis is needed here. It remains possible that cell division might cheat the turgor force in Gram-negative bacteria, but would have to constrict against the full and much larger turgor force in Gram-positive bacteria.

Here we would like to discuss three potential sources for constriction force, and how they might function even in the presence of turgor force: (A) FtsZ bending the PM from the inside; (B) peptidoglycan assembly pushing the PM from the outside; and (C) excess membrane synthesis pushing the membrane to expand inward. The idea of membrane pushing suggests a new force mechanism in which the combined force of FtsZ pulling and membrane pushing could divide bacteria even against turgor pressure.

***(A) FtsZ bending the PM from the inside:*** A number of mechanisms have been proposed for how FtsZ might generate a constriction force, which we have discussed previously (Erickson, 2009; Erickson et al., 2010; Erickson and Osawa, 2017). We favor a model where curved protofilaments generate a bending force on the membrane (Osawa et al., 2009). The strongest evidence came from experiments where FtsZ was tethered to liposomes by an artificial membrane tether. With the tether [(mts) membrane targeting sequence] on the normal C-terminal side, FtsZ-mts assembled Z rings inside tubular liposomes that constricted from the inside (Osawa et al., 2008). When the tether was switched to the opposite side, mts-FtsZ assembled Z rings on the outside, and these constricted the liposomes by squeezing (Osawa and Erickson, 2011). See Supplementary Figure S1 in Supplemental Material for images and diagram. These results are consistent with the FtsZ protofilaments having a defined curvature, and the direction of force determined by whether the tether was on the outside or inside of the curve.

**FIGURE 1:**
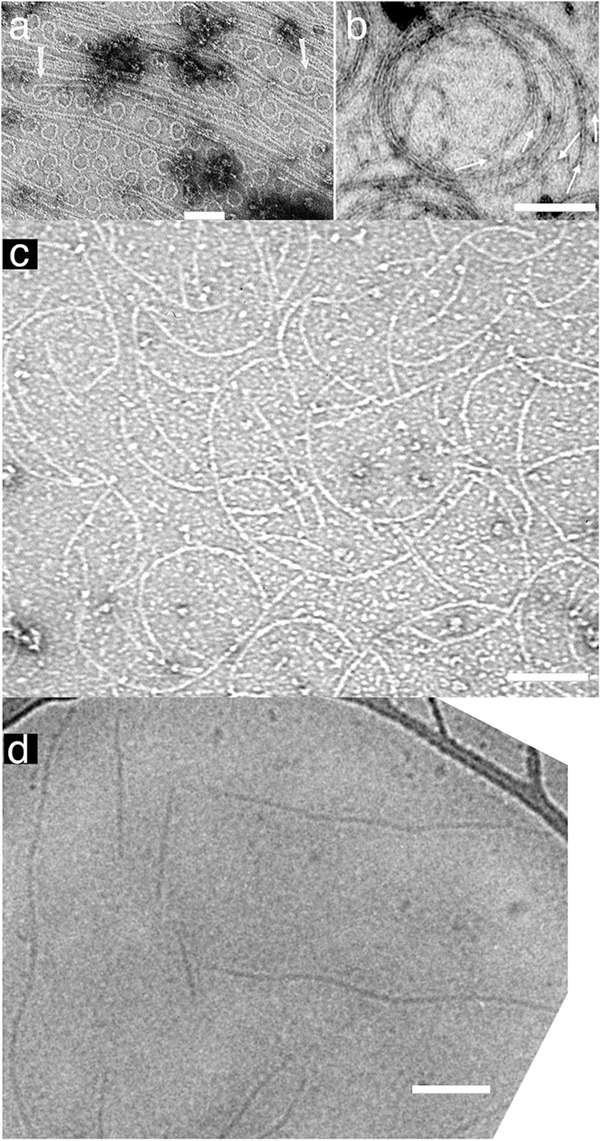
Straight and curved protofilaments. **(a)** FtsZ protofilaments were absorbed on a cationic lipid membrane and then negatively stained for observation by EM. Many minirings approximately 24 nm diameter were observed. These minirings were probably stabilized with GDP-FtsZ (reprinted from Erickson et al., 1996). **(b)** FtsZ was polymerized into protofilaments in crowding buffer solution and then negatively stained. The protofilaments attached loosely to each other and formed curved bundles (inner diameter of the toroid is approximately 200 nm) (reprinted from Popp et al., 2009). **(c)** Protofilament assembly was observed without Mg^2+^. In this condition all subunits in protofilaments were FtsZ-GTP. *Pseudomonas aeruginosa* FtsZ was used in this image (micrograph courtesy of Dr. Yaodong Chen, Northwest University, Xi’an, China). **(d)** FtsZ was polymerized into protofilaments in solution and then observed by cryo EM. These protofilaments, which apparently made no lateral contact with each other or a substrate, appear straight with some wavy structure (reprinted from Turner et al., 2012). Bars are 100 nm.

A curved protofilament conformation was first observed for *E. coli* FtsZ adsorbed to cationic lipid monolayers (**Figure 1a**) (Erickson et al., 1996). These 24 nm diameter minirings were favored by GDP, suggesting a hydrolysis-linked conformational change. However, they have only been found for FtsZ from a few bacterial species (discussed in Erickson and Osawa, 2017). Also, GTPase deficient FtsZ can function in suppressor strains, suggesting that GTP hydrolysis is not essential for force generation and cell division (Bi and Lutkenhaus, 1992; Osawa and Erickson, 2006).

There is an alternative curved conformation that we have called “intermediate curved” (Erickson et al., 2010; Erickson and Osawa, 2017) with a diameter 100–200 nm. In many cases, these curved filaments appear mixed with straight protofilaments by negative stain EM, but they were more prominently developed after adsorption to a mica surface (Mingorance et al., 2005; Hamon et al., 2009; Mateos-Gil et al., 2012) or in crowding conditions (Popp et al., 2009) (**Figure 1b**). Intermediate curved protofilaments could also be assembled in GTP plus EDTA, where the absence of Mg completely blocks GTP hydrolysis (Chen et al., 2005) (**Figure 1c**). Intermediate curved filaments were observed in a reconstitution system *in vitro* with FtsZ-mts (Osawa et al., 2009) or FtsZ/FtsA on a supported lipid bilayer (Szwedziak et al., 2014). Furthermore, the diameter of this curvature fits ∼200–300 nm minimum diameter of the Z ring *in vivo* (Soderstrom et al., 2014; Coltharp et al., 2016). We consider this intermediate curvature the likely conformation that generates bending force on the membrane (Erickson and Osawa, 2017).

Two recent studies have shown FtsI moving in circular paths around the Z ring, driven by patches of treadmilling FtsZ (Bisson-Filho et al., 2017; Yang et al., 2017). This may be important for distribution of the CW remodeling molecules, but may not be essential for constriction, because the treadmilling is governed by GTP hydrolysis which is not essential for division.

If GTP hydrolysis is not involved, then what drives the conformational change from straight to curved? One possibility is that the protofilaments are intrinsically curved and may bend the membrane like Bar domains in eukaryotic systems (Saarikangas et al., 2009). Another possibility is a transition from twist to curvature. Although FtsZ protofilaments with no attachment to any surfaces appear mostly straight in cryo EM (**Figure 1d**) (Turner et al., 2012), those filaments might be naturally twisted. Previous studies have suggested that the protofilament conformation might be a balance of twist and curvature (Arumugam et al., 2012; Gonzalez de Prado Salas et al., 2014). When a twisted filament binds the membrane the twist must straighten (so that all subunits face the membrane) and the energy is transferred to a curvature. Lateral interactions of several types seem to induce the curved conformation, perhaps by forcing untwisting (Mingorance et al., 2005; Hamon et al., 2009; Popp et al., 2009; Mateos-Gil et al., 2012). This hypothesis seems to be contradicted by the existence of straight, untwisted protofilaments in crystals of *Staphylococcus aureus* FtsZ (Matsui et al., 2012), but it remains an interesting idea.

**FIGURE 2:**
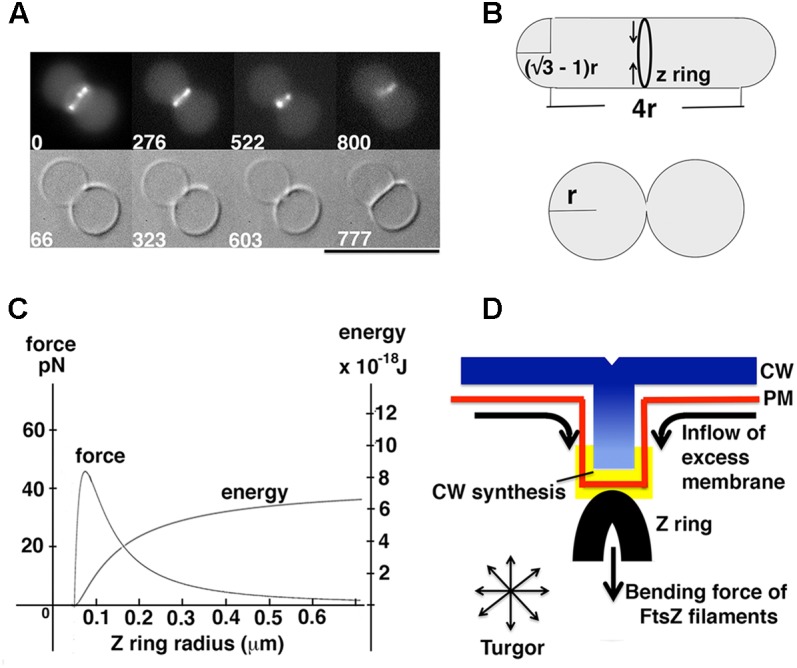
Model for force generation by the Z ring *in vitro* and *in vivo*. **(A)** FtsZ formed Z rings in unilamellar liposomes with FtsA^∗^ (R286W), and the reconstituted Z rings divided liposomes with a clear septum (reprinted from Osawa and Erickson, 2013). A constraint on liposome division is posed by the fact that the membrane cannot be stretched more than 4% of the original area (Needham and Nunn, 1990). Because the membrane is impermeable to ions, reducing the volume by removing water would lead to higher salt inside and consequent osmotic pressure. With these constraints one cannot divide a spherical liposome, because the two daughter liposomes would have to shed 20% of the water of the mother. **(B)** Schematic view of liposome division by Z rings. In this particular geometry surface area and volume of the liposome does not change after division, in which case the Z ring only needs to bend the membrane. The successful division of the liposome in **(A)** probably approximates this geometry. **(C)** The relationship between force and energy with different Z ring radius. The graph was made from the equations for bending an isotropic rod (Supplementary Material) (Lan et al., 2009). **(D)** A model showing how excess phospholipid production can play a key role in Z ring constriction.

***(A1) Estimation of energy and force generated by Z rings:*** Knowing that reconstituted Z rings can divide unilamellar liposomes (**Figure 2A**), a minimum requirement of energy and force for liposome division can be estimated. Certain geometries, such as that shown in **Figure 2B** (Supplementary Material), generate daughters with surface area and volume equal to the mother. We assume that successful divisions of liposomes *in vitro* (**Figure 2A**) (Osawa and Erickson, 2013) approximate this geometry.

To estimate the minimum energy needed to achieve liposome division, we used Helfrich’s equation (Helfrich, 1973), and calculated the bending energy of the membrane before and after division (**Figure 2B**). The derivation is given in Supplementary Material. A second calculation gave the minimum initial constriction force that could be generated by a Z ring based on the bending energy/force of isotropic rods (Lan et al., 2009). The minimum energy for full constriction of the liposome shown in **Figure 2B** is 8.0 × 10^-19^ J, which also estimates 0.35 pN as the minimum initial constriction force for a liposome of 1 μm diameter. The second calculation gives 5.6 × 10^-18^ J and 2.45 pN as the potential energy and force that could be generated by FtsZ filaments for initial constriction of a 1 μm diameter bacterium. These are similar to values described previously with different parameters (Lan et al., 2009). Both the energy and force for FtsZ bending are about 10 times larger than the minimum requirements for liposome division, consistent with the observed constriction *in vitro* (Osawa and Erickson, 2013). As shown in **Figure 2C**, the constriction force actually increases up to 45 pN at 200 nm diameter, which is approximately where FtsZ leaves the Z ring (Soderstrom et al., 2014; Coltharp et al., 2016).

In the absence of turgor, this force would be sufficient to divide a liposome of appropriate geometry. FtsZ bending membranes is almost certainly the major source of constriction force in mycoplasma and some archaea, which lack a CW and have minimal turgor force. If excess membrane were provided it could divide almost any geometry. However, it would probably not be sufficient to constrict the PM in the face of strong turgor.

***(B) Peptidoglycan assembly pushing the PM from the outside:*** Another source of constriction force could be inward growth of the CW. Because the CW is a rigid structure, it should be capable of supporting the PM even if it is pressing against it with full turgor pressure. Coltharp and colleagues have suggested recently that the inward growth of the CW should be the major source of constriction force, while FtsZ serves primarily as a scaffold to define the location and orientation of the CW remodeling proteins (Coltharp et al., 2016; Coltharp and Xiao, 2017). In this case the curvature of FtsZ filaments may just work for fitting to the diameter of constriction site.

A detailed mechanism of pushing by CW ingrowth has not yet been developed. If the CW is to push the PM, it would seem necessary for it to contact the PM directly or indirectly through a membrane protein. This would need to eliminate the periplasmic space at the site of the site of contact. The invaginating CW must then insert glycan strands against the force on the PM. This would likely require a Brownian ratchet mechanism, in which either the CW or the PM were moving with sufficient distance and frequency to permit the glycan strands to slip in. In models of actin pushing membranes it was found that a simple Brownian ratchet would not work. The key was an elastic Brownian ratchet, in which flexing of the actin filaments provided the gaps for insertion (Mogilner and Oster, 1996). It is not clear where the Brownian motion would occur for CW pushing the PM against turgor. This may not work.

Plant and yeast cells are in a similar situation where the PM is surrounded by a rigid CW and is exposed to turgor pressure (Tepfer and Taylor, 1981; De Nobel et al., 1989). A recent paper proposed that the actin-myosin contractile ring for yeast division cannot constrict against this high turgor pressure (Proctor et al., 2012). This study found that constriction often continued after disruption of the actin-myosin ring, and concluded that the major constriction force is generated by the CW invagination.

Another consideration is that both plant and yeast cells can achieve endocytosis with an intracellular apparatus made of protein molecules (Samaj et al., 2005; Goode et al., 2015). This indicates that intracellular proteins can deform the membrane against high turgor pressure, without any contribution from CW invagination. It is not known how this works, but the mechanism may apply also to how bacteria divide even under turgor pressure. We suggest that the mechanism may utilize force from excess membrane synthesis, discussed next.

***(C) Excess membrane synthesis pushing the membrane to expand inward:*** During cell elongation, membrane synthesis must match the expansion of the CW. If, however, membrane is synthesized in excess, it could only be accommodated by blebbing of the membrane. For a bacterial, plant or yeast cell, where the membrane is trapped by the rigid CW, the membrane can only bleb inward. Importantly, if a bleb is initiated by even a small force from a Z ring (or an endocytic apparatus), the excess membrane synthesis may contribute to further extension, perhaps even matching the turgor pressure (**Figure 2D**).

The extension of the rod during bacterial elongation is constant in both *E. coli* and *B. subtilis*, but this extension is reduced during Z ring constriction in both species (Burdett et al., 1986; Coltharp et al., 2016). If membrane synthesis continues at the same rate, it would be produced in excess during the constriction phase. One study actually showed that phospholipid production increased in both species during cross wall formation (Carty and Ingram, 1981), although another study of *E. coli* suggested that the productions of CW and phospholipid are synchronized (Gally et al., 1993). These studies were done 20–30 years ago and there has been no update. Because phospholipids are produced in the PM in bacteria (Zhang and Rock, 2008), phospholipid synthesis directly increases the area of PM and may largely cancel the effect of turgor pressure pressing the PM against the CW, especially once the PM initiates bending.

Because of the constraining CW, excess phospholipid production must push the membrane inward. A possible example is in Gram-positive *Streptomyces* species that grow as vegetative hyphae, where the PM can invaginate and form membrane cross walls without Z ring or CW support (Celler et al., 2016). This suggests that membrane production may be sufficient for membrane invagination against turgor pressure [However, the existence of cross walls in *Streptomyces* lacking FtsZ has been questioned by a recent study (Santos-Beneit et al., 2017)]. Membrane septa without CW were also observed in *B. subtilis* when one peptidoglycan synthase, PBP2B, was deleted (Daniel et al., 2000). Similar membrane septa were observed in *E. coli* with deletion of several lytic hydrolases (Heidrich et al., 2002), or with mutations in FtsK (Berezuk et al., 2014). Internalized PM was also observed in *B. subtilis* when membrane was overproduced by overexpression of AccDA, the enzyme for fatty acid synthesis (Mercier et al., 2013). That study also made a strong case that excess membrane synthesis was necessary and sufficient for division of *B. subtilis* L forms, lacking the CW and FtsZ. We suggest that septum formation without CW invagination is probably driven by excess membrane pushing the membrane inward, in some cases directed and assisted by the constriction force of the Z ring.

### Combined Forces of FtsZ Pulling and Membrane Pushing May Divide Bacteria Even under Turgor Pressure

Assuming a substantial contribution from excess membrane production, the requirement of force generation by Z rings can probably be much smaller than expected previously (Lan et al., 2007). If excess membrane is exerting a lateral compressive force on the PM, a bleb may be initiated with only a small invagination force from FtsZ. A force of 2.45 pN or even 0.35 pN may suffice, depending on factors such as the pushing force of membrane and the initial bending curvature of membrane. Continued membrane synthesis would add substantially to any invagination force.

Excess membrane production may also facilitate CW ingrowth by canceling the effect of turgor pressure that pushes the PM against the ingressing CW. This could facilitate or eliminate the need for a Brownian ratchet to insert new glycan strands. In this case CW production might contribute to constriction by working as a ratchet to stabilize the newly constricted membrane. This could also explain how CW synthesis could be rate limiting for constriction (Coltharp et al., 2016). Bisson-Filho et al. (2017) suggested that dynamic FtsZ filaments may deform the membrane, and PG synthesis could reinforce the deformation on the other side. The PG reinforcement could be rate limiting.

The force generated by membrane synthesis could be sufficient to power invagination against a turgor force. Even in Gram-negative bacteria, whose PM is thought to be in an isoosmotic balance between cytoplasm and periplasm, membrane synthesis is needed to keep up with the invagination, and to avoid developing turgor pressure as cytoplasmic volume is reduced by septal ingrowth. This proposal might be also extend to yeast division and to endocytosis in yeast and plant cells, with excess PM providing an essential part of the force for membrane invagination.

### Future Directions

A major problem is lack of information about the osmolarity of the periplasmic space in Gram-positive bacteria. Without this we don’t even know whether cell division needs to overcome turgor pressure. Important new tools would be bacterial strains where FtsZ bending, CW synthesis and membrane synthesis can be regulated up and down. Biosensors that can measure the force generated by FtsZ and CW would be especially useful.

## Author Contributions

MO wrote and performed the theoretical analysis; HE assisted writing and deeply discussed a wide range of problems.

## Conflict of Interest Statement

The authors declare that the research was conducted in the absence of any commercial or financial relationships that could be construed as a potential conflict of interest. The reviewer MF and handling Editor declared their shared affiliation.
