# Supplementary material for: Turgor Pressure and Possible Constriction Mechanisms in Bacterial Division
Source: Front Microbiol. 2018 Jan 31;9:111. doi: 10.3389/fmicb.2018.00111 (PMC5797765; doi:10.3389/fmicb.2018.00111)
Supplement: Supplementary file 1 [file Presentation_1.pdf]

Supplementary Material

Turgor pressure and possible constriction mechanism in bacterial division.

Masaki Osawa*, Harold P. Erickson

Correspondence: Masaki Osawa: masaki.osawa@duke.edu

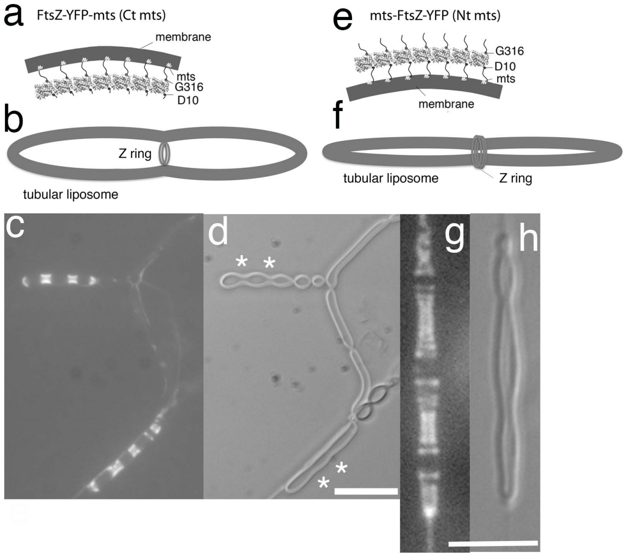

Fig. S1 Reconstitution of Z rings with membrane targeted FtsZ. (a) Schematic view of how the FtsZ-YFP-mts filament binds a membrane. The C-terminal mts is on the outside of the curved protofilament, and binds to concave membranes. (b) Schematic view of reconstituted Z ring with FtsZ-YFP-mts inside a tubular multilamellar liposome. (c, d) Reconstituted Z rings inside tubular multilamellar liposomes; (c) is the fluorescence image of FtsZ, and (d) is the DIC image of the liposome. These are the very first reconstituted Z rings we observed in liposomes. The rings generated constrictions (*). (e) Schematic view of how the mts-FtsZ-YFP filament binds a membrane. The N-terminal mts is ~180 degree from the C terminus, placing it on the inside of the curved

protofilament. mts-FtsZ-YFP filaments bind convex membranes. (f) Schematic view of Z rings formed on the outside of tubular liposomes with mts-FtsZ-YFP. (g-h) Image of Z rings formed on the outside of tubular liposomes with mts-FtsZ-YFP. Bars are 10 μm for (c, d) and 5 μm for (g,h).

Calculation of the minimum force needed to constrict a liposome in vitro, and the force that can be generated by FtsZ protofilament bending in bacteria.

One constraint for constriction of a liposome is that the volume of the daughter cells cannot be smaller than that of the mother cell. This is due to the lack of stretch in the membrane and strong osmotic pressure due to the impermeability to ions. Another constraint may be imposed by the agarose in which the liposome is embedded. Agarose was used to immobilize the liposomes for imaging, but a potential disadvantage is that even a soft agarose gel may resist the shape change of the liposomes. However, small liposomes (diameter < 5 μm) may be able to change their shape without this pressure, since a soft agarose gel made with high salt buffer has a random shape of large pores (Maaloum et al., 1998). Although Z rings reconstituted with FtsA can sometimes divide liposomes, in many cases we observed that the Z rings halted the constrictions. We think that this arrest may be due either to decrease in volume or resistance from the agarose gel.

Nevertheless, if we focus on the smaller fraction of constrictions that proceed to division, which apparently avoid these constraints, we can calculate the minimum energy requirement and corresponding force for liposome division.

What is the minimal force needed to constrict a liposome in vitro?

To estimate the minimum force generation needed for this division, we calculated the difference between the bending energy of the membrane before and after division shown in Fig. 2b (in the main manuscript), assuming that the energy for division depends only on the bending energy of the membrane. We use Helfrich's equation as shown below (Helfrich, 1973).

$$E_{bend}M = \int dA\{\frac{1}{2}\kappa\left(\frac{1}{R_1}+\frac{1}{R_2}-\frac{2}{R_0}\right)^2+\kappa'\left(\frac{1}{R_1R_2}\right)\} \qquad \text{------(1)}$$

where $E_{bend}M$ is the free energy for bending the membrane, A is the surface area of membrane, R_1 and R_2 are the principle curvature radii of the capsule shape (Fig. 2b, main text), R_0 is the preferred curvature radius, κ is the membrane bending rigidity and κ' is a Gaussian curvature modulus which is only effective when a topology change occurs. The term including κ' is ignored because there is no topological change in the constriction process (Almendro-Vedia et al., 2013). For this we assume that Z rings constrict to a radius of 50 nm, which is the smallest radius for intermediate curved FtsZ filaments described above, and the smallest radius in the reconstituted Z rings imaged by cryoEM (Szwedziak et al., 2014) . It is also approximately the radius at which FtsZ dissociates from the constriction (Soderstrom et al., 2014, Coltharp et al., 2016). The minimum energy that Z rings need to divide the liposome can be calculated as the difference in $E_{bend}M$ after and before constriction.

The $E_{bnd}M$ after constriction is approximated with the two spheres of radius r, as shown in Fig. 2b. For $E_{bend}M$ of a sphere the area A is $4\pi r^2$, where R_1 and R_2 are both = r, and $R_0 = \infty$. The result should be multiplied by 2 because 2 spheres are produced by constriction. Therefore, $E_{bend}M$ (after constriction) = $16\pi\kappa$.

For the liposome before division we calculate $E_{Bend}M$ separately for the cylindrical part and the hemispherical caps. The radius in all cases is (√3-1) r for the particular geometry shown in Fig. 2b, where r is the radius of the two spheres after division. The length of the cylinder is 4r. For the cylinder R_1 = (√3-1) r, $R_2 = R_o = \infty$, giving $E_{bend}M$ = 4πκ/(√3-1). Because both ends of cylinder are capped by hemispheres, 8πκ for a sphere was added. Therefore $E_{bend}M$ (before constriction) = 8πκ + 4πκ/(√3-1). Note that the $E_{bend}M$ before and after do not depend on r, indicating that the liposome size does not affect the bending energy value.

The minimum energy for full constriction of Z ring for this liposome is 16πκ - [8πκ + 4πκ/(√3-1)] = 8πκ - 4πκ/(√3-1). Using $\kappa = 10^{-19}$ J, which is the value for general membrane bending rigidity (Dimova, 2014), the constriction energy is 8.0 x 10^{-19} J.

I now calculate how much force a Z ring would need to achieve this energy. I start with the following equation for the bending energy of isotropic rods (Lan et al., 2009).

$$E_{bend}Z = L\frac{1}{2}\kappa_z\left(\frac{1}{R_3} - \frac{1}{R_{z0}}\right)^2 \quad \text{----------(2)}$$

where L is the total length of all FtsZ filaments in the Z ring, κ_z is the bending rigidity of the FtsZ filament, R_3 is actual curvature radius of the FtsZ filament and R_{Z0} is the preferred curvature radius. The force for this Z ring is obtained by differentiation by R_3, which gives

$$ForceZ = L\kappa_z\left(\frac{1}{R_{z0}} - \frac{1}{R_3}\right)\left(\frac{1}{R_3}\right)^2 \quad \text{----------(3)}$$

Combining equations (2) and (3) we can express ForceZ as a function of $E_{bend}Z$, with no explicit dependence on L and κ_z

$$ForceZ = \frac{2 \times E_{bend}Z}{\left(\frac{1}{R_{z0}} - \frac{1}{R_3}\right) R_3^2} \quad \text{-------(4)}$$

To obtain the minimum force for the Z ring constriction in liposomes, we now assume that $E_{bend}Z$ will be equal to value of $E_{bend}M$, which was estimated above from the geometry of the liposome before and after constriction. We therefore set $E_{bend}Z$ = 8.0 x 10^{-19} J; R_3 = 500 nm, which is the radius of a bacterium before constriction; and R_{z0} = 50 nm, which is the preferred radius of the intermediate curved filament. This minimum constriction force needed at the beginning of constriction is 0.35 pN.

What is the force that curved FtsZ filaments can generate?

As an alternative calculation of the energy and force, I use equations (2) and (3), which are based only on the mechanical rigidity of FtsZ. The bending modulus κ of an FtsZ filament is Lp k_B T, where Lp is the persistence length of FtsZ filaments, k_B is Boltzmann's constant, and T is absolute temperature. Turner et al measured Lp = 1.15 μm using cryo EM (Turner et al., 2012). The total length of all FtsZ filaments in the Z ring, L, is 7.2 μm, which is from a simple calculation: 4 nm (size of FtsZ) x 6000 (number of FtsZ in a cell) x 0.3 (fraction of FtsZ in the Z ring). R_{z0} and R_3 are 50 and 500 nm, respectively, as described above. Then the energy and initial force are obtained as 5.6 x 10^{-18} J and 2.45 pN, respectively. Both the energy and force here are about 10 times larger than that calculated as the minimum required to divide the unilamellar liposome in vitro. This is consistent with the observation that FtsZ plus FtsA can easily divide liposomes in vitro, and suggests that the constriction force of FtsZ is more than sufficient

to constrict the membrane of cells, provided there is minimal turgor force, and/or that there is excess membrane available. Importantly, this number is similar to that determined previously by Lan et al using a similar calculation (Lan et al., 2009).

References

ALMENDRO-VEDIA, V. G., MONROY, F. & CAO, F. J. 2013. Mechanics of constriction during cell division: a variational approach. *PLoS One,* 8**,** e69750.

COLTHARP, C., BUSS, J., PLUMER, T. M. & XIAO, J. 2016. Defining the rate-limiting processes of bacterial cytokinesis. *Proc Natl Acad Sci U S A,* 113**,** E1044-53.

DIMOVA, R. 2014. Recent developments in the field of bending rigidity measurements on membranes. *Adv Colloid Interface Sci,* 208**,** 225-34.

HELFRICH, W. 1973. Elastic properties of lipid bilayers: theory and possible experiments. *Z Naturforsch C,* 28**,** 693-703.

LAN, G., DANIELS, B. R., DOBROWSKY, T. M., WIRTZ, D. & SUN, S. X. 2009. Condensation of FtsZ filaments can drive bacterial cell division. *Proc Natl Acad Sci U S A,* 106**,** 121-6.

MAALOUM, M., PERNODET, N. & TINLAND, B. 1998. Agarose gel structure using atomic force microscopy: gel concentration and ionic strength effects. *Electrophoresis,* 19**,** 1606-10.

SODERSTROM, B., SKOOG, K., BLOM, H., WEISS, D. S., VON HEIJNE, G. & DALEY, D. O. 2014. Disassembly of the divisome in Escherichia coli: evidence that FtsZ dissociates before compartmentalization. *Mol Microbiol,* 92**,** 1-9.

SZWEDZIAK, P., WANG, Q., BHARAT, T. A., TSIM, M. & LOWE, J. 2014. Architecture of the ring formed by the tubulin homologue FtsZ in bacterial cell division. *Elife,* 3**,** e04601.

TURNER, D. J., PORTMAN, I., DAFFORN, T. R., RODGER, A., ROPER, D. I., SMITH, C. J. & TURNER, M. S. 2012. The Mechanics of FtsZ Fibers. *Biophysical journal,* 102**,** 731-8.
